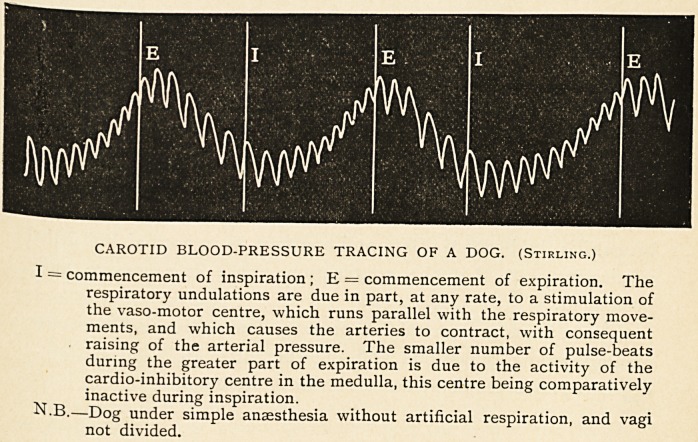# On the Probable Rhythmical Contraction of the Bronchial Muscular Coat as a Factor in Pulmonary Disease

**Published:** 1903-03

**Authors:** P. Watson Williams

**Affiliations:** Lecturer on Practical Medicine, University College, Bristol; Assistant-Physician, Bristol Royal Infirmary.


### ON THE PROBABLE RHYTHMICAL CONTRACTION OF THE BRONCHIAL MUSCULAR COAT AS A FACTOR IN PULMONARY DISEASE.

BY

P. Watson Williams, M.D. Lond.,

*Lecturer on Practical Medicine, University College, Bristol; Assistant-Physician, Bristol Royal Infirmary.*

---

A consideration of the pathology of certain pulmonary affections led me, some years ago, to the view, which I expressed in an article on Bronchitis in the *Encyclopædia Medica* [1]:—"It is not improbable that, like the alæ nasi and vocal cords, the bronchial muscle may rhythmically dilate and contract with inspiration and expiration. I believe that this largely explains why in bronchitis and asthma the dyspnœa is expiratory rather than inspiratory, inasmuch as the muscular spasm is more or

[1] Vol. ii., 1899, p. 117.

less inhibited during inspiration, permitting air to be inspired more freely than during the period of bronchial spasm it can be expired, consequently the lungs become distended and emphysematous." I was led to the conviction that such rhythmical contractions occurred as the only satisfactory explanation of the pulmonary distension in bronchitis and asthma, although I found no mention of anything of the kind in the current text books of physiology, pathology, or medicine; but more extended search has shown me that in postulating this rhythmical action I am only reviving an obsolete and abandoned theory.

Certain **clinical phenomena** associated with asthma, bronchitis, and emphysema afford strong evidence in support of the theory of rhythmical bronchial contractions and dilatations, without which no satisfactory explanation is forthcoming.

(1.) The theory that spasmodic asthma is due to spasm of the muscular coat of smaller bronchi—first put forward by Reisseissen, and upheld by Hyde Salter—is that which finds most general acceptance at the present time. Indeed, Dr. Goodhart [1] states that "this view, originally affirmed by Reisseissen, . . . has since then been proved on experimental observation by numerous observers from C. J. B. Williams onwards, including Paul Bert, Riegel, Biermer, Lazarus, and others."

It is said that, the bronchial obstruction being incomplete, the air enters the lungs under the labour of forced inspiration, but that expiratory paralysis or obstruction prevents its getting out: that "the spasm of the bronchial tubes must tend to prevent air getting in and out; and the more in or out according as the inspiratory or expiratory force is the greater.[2]

But in asthma the dyspnœa is mainly expiratory; and it has been proved that the expiratory force in forced respiration is much greater than the inspiratory, and the difficulty of reconciling these facts with the occurrence of over-distension of the lungs in asthma has given rise to much controversy; indeed, so great is the difficulty of accepting the bronchial spasm theory that Wintrich and others have fallen back on the theory that asthma is due to spasm of the diaphragm, while

[1] Allbutt's *System of Medicine*, vol. v., 1898, p. 298.

[2] Goodhart, *loc. cit.*

Weber and his followers have introduced the hypothesis that asthma is due to vascular engorgement of the bronchial mucosa. None of these theories are satisfactory or sufficiently supported by clinical observation to meet with general acceptance, hence it may be said that no theory has yet been put forward which is not open to obvious criticism.

But I venture to suggest that if we admit that the bronchi dilate on inspiration and contract on expiration, all the difficulties in accounting for the clinical phenomena alluded to at once disappear. Instead of having to hypothecate that asthma "contravenes usual rules," or that "natural order is destroyed," we see that the contraction phase of physiological respiration is merely accentuated; consequently, expiration is so prolonged that a fresh inspiration has to be made before the expiration is complete, until at length, the chest becoming more and more distended, there is no possibility of distending the chest any further, however forcible the inspiratory effort may be.

(2.) In acute bronchitis, again, we find certain phenomena which are explicable by a rhythmical contraction of the bronchi, viz., the prolongation of expiration, and in marked dyspnœa the tendency to over-distension of the lungs, in conjunction with patches of collapsed lung when any small bronchial tube has been occluded by mucus which has been sucked down. Over-distension of the lungs (acute emphysema) is most obvious in the acute suffocative bronchitis of the small tubes, in which more air is at first drawn in by the forced inspiration than can be expelled by the expiration, much prolonged as it is. In capillary bronchitis of young children, on the contrary, the bronchial muscular coat is prone to early paresis or paralysis, with resulting acute bronchiolectasis[1]

(3.) The development of acute emphysema and chronic hypertrophous emphysema appears to me capable of more satisfactory explanation by excessive contraction of the bronchioles during expiration, than by any current hypothesis. Laennec was the first to establish the now abandoned theory that chronic emphysema is due to distension of the lungs during inspiration, the

[1] Cf., "Bronchiectasis in Young Children," J. W. Carr, *Practitioner*, 1891, xlvi. 86; "Acute Bronchiectasis," S. J. Sharkey, *St. Thomas's Hosp. Rep.*, 1894, xxii. 33; Wilson Fox, *Diseases of the Lungs and Pleura*, 1891, p. 114.

air thus inspired being unable to escape during expiration, owing to obstruction in the bronchi from catarrhal swelling or accumulated mucus. Sir William Jenner's expiratory hypothesis was stated in 1857, and is still generally accepted. In short, his theory was that frequently-recurrent cough, with consequent compression of the lungs when the glottis was closed, results in the distension of the air vesicles; and he made a great point of the fact that the portions of the lungs which are least supported by the chest walls are, as we should expect, just the parts where hypertrophous emphysema begins. This, however, does not account for the fact that, sooner or later, large lunged emphysema becomes general throughout the lung; and he had to introduce the hypothesis that the lungs yielded in time in portions corresponding with the intercostal spaces, and that as the lung became enlarged by emphysema it shifted its position in relation to the chest wall, and so successive portions corresponded with the intercostal spaces.

Now the chest walls are the most unyielding compressors of the lung in coughing, and thus the greater portion of each lung which is supported and compressed should be collapsed rather than emphysematous, *i.e.* if Jenner's theory is the correct solution; whereas, so far from this being the case, even the chest wall itself becomes distended in chronic emphysematous persons. Instead of the cough, I suggest that the cause of the emphysema is the recurrent attacks of bronchitis or the persistence of conditions causing difficult and prolonged expiration, and therefore chronic over-distension of the lung. An exactly similar process results in the chronic emphysema of chronic asthmatics. If constant cough was really the cause of hypertrophous emphysema, is it not curious that this form of emphysema is so remarkably rare in phthisical patients, who, above all others, are the subjects of frequent cough, persisting for years?

Indeed, violent and persistent cough may be said *per se* to cause pulmonary collapse rather than emphysema, and an excellent illustration of this fact was afforded by a young child recently admitted to the Royal Infirmary under Dr. Waldo, suffering from whooping cough. On admission, as the result of violent paroxysms of cough, very extensive collapse of the lung was found to exist; but this soon disappeared by the spontaneous distension of the collapsed areas. Surely if violent cough is the cause of emphysema, this child should have been the subject of acute emphysema, and the fact that the chest walls were so yielding should have favoured its occurrence if Jenner's theory is correct.

The **physiological evidence** tending to support the theory of rhythmical bronchial contractions is indirect, inasmuch as no observer has yet succeeded in conclusively demonstrating these contractions and dilatations corresponding with the respiratory movements. But it is obvious that there are many inherent difficulties in making correct observations of the slight contractions and dilatations of the deep-lying bronchioles, where alone broncho-motor phenomena, if they exist, would be most pronounced. In quiet respiration, at any rate, bronchiole movements must be very slight, and the fact that the respiratory movements of the alæ nasi and of the vocal cords, which are so pronounced in the quiet respiration of infants, are often entirely in abeyance in adults, points to the conclusion that these broncho-motor phenomena are most marked in early life, or in dyspnœa. Nevertheless, the indirect evidence of these rhythmical bronchial contractions appears to be fairly conclusive.

If in a dog one vagus nerve in the neck be divided and the central end stimulated, the bronchi of both lungs contract. If, however, the dog be deeply anæsthetised by ether, stimulation of the cut vagus (central end) causes dilatation of the bronchi. The experimental evidence on this point was sufficiently convincing to lead to the statement by Landois and Stirling that "it seems plain that the vagi contain centripetal or afferent fibres, which can cause both expansion and contraction of the bronchi."[1]

With these facts admitted, it has yet to be shown that the bronchial dilatation and contraction is *rhythmical*, and synchronises with inspiration and expiration.

(1.) It is generally conceded by physiologists that the

1 *A Text-Book of Human Physiology*, 4th ed., vol. i., 1891, p. 189.

respiratory centre consists practically of two centres alternately in activity—an inspiratory centre and an expiratory centre; and, further, that running in the pneumogastric nerves are accelerating or inspiratory fibres, which normally come into action when the lungs are diminished in volume, *i.e.* at the end of expiration, and inhibitory or expiratory fibres, which normally come into action when the lungs are expanded at the end of inspiration. But none of the physiological text books, as far as I know, refer to a corresponding rhythmical dilatation and contraction of the bronchi (which seems a natural inference), with the exception of Foster,[1] who states that it is uncertain whether there exists a rhythmic contraction of the trachea and bronchial passages effected by means of the muscular coat, and synchronous with the respiratory movements of the chest.

(2.) The movements of the vocal cords during respiration are not without significance, for the glottic aperture may be regarded as a portion of the respiratory tract, more highly specialised for the purposes of phonation. This statement is supported by the fact that in birds the specialised phonatory portion of the respiratory tract is lower down at the bifurcation of the bronchi. The glottis manifestly dilates and contracts during inspiration and expiration, and in children even during quiet respiration. It is worthy of note here that experiments by Hooper, of Boston, and by Semon and Horsley agreed in showing that when various animals were deeply narcotised by ether, stimulation of the peripheral end of the divided (as well as of the undivided) recurrent nerve resulted invariably in abduction of the vocal cords, in contra-distinction to the adduction which was invariably produced by the similar stimulation of the recurrent in animals not deeply under the influence of anæsthetics.

Further, the close analogy between the glottic muscles and those of the bronchi is suggested by Rosenthal's observations,[2] that the inhibitory nerves, which affect the respiratory centre, run in the superior laryngeal nerve, and also in the recurrent laryngeal, to the respiratory centre. The superior laryngeal nerve contains

[1] *A Text-Book of Physiology*, 6th ed., part ii., 1895, p. 578.

[2] Landois and Stirling, *op. cit.*, vol. i., 1891, p. 821.

a large number of inhibitory respiratory nerves, or nerves which cause arrest of the respiration in expiration. We become aware of this when a foreign body enters the larynx, the strong expiratory effort and laryngeal spasm causing cough and expulsion of the body. On the other hand, the main trunk of the vagus contains afferent fibres, stimulation of which causes acceleration of respiration with strong inspiratory effort, and which may be arrested in the position of extreme inspiration.

*Since stimulation of the expiratory fibres and the phase of extreme expiration is known to be attended with bronchial contraction, it seems probable that the stimulation of inspiratory or accelerating fibres and the phase of inspiration is attended with bronchial dilatation.*

(3.) If vagi nerve fibres do cause dilatation and contraction of the bronchial tubes, synchronous with inspiration and expiration, we should find some evidence of such rhythmic bronchial movements if the vagi are cut. Hence, after section of both vagi inspiration is long and deep, but expiration is short and active, and is followed by a pause, so quickly is expiration effected; whereas before section the inspiration is shorter than expiration. Some factor which caused expiration to be prolonged as compared with inspiration, has been removed by section of the vagi, and I suggest, as the explanation, that before section of the vagi the smaller bronchi dilate on inspiration, and contract on expiration. This would cause a higher air tension in the alveoli to be maintained, and, as a consequence, the *rate* of oxygen and carbonic-acid disassociation to proceed more rapidly than it would if the inspired air was emptied out of the chest as readily and quickly as it entered. Hence we hear normally the short inspiratory vesicular murmur during the whole inspiration, but the expiratory vesicular murmur only towards the commencement of expiration, for after bronchiole contraction the air escapes so slowly that, except in pathological conditions, no vesicular murmur is audible.

While such rhythmic contractions of the bronchioles, like those of the glottis, are probably slight and relatively unimportant in quiet respiration, they would obviously be more pronounced and of greater physiological value during forced respiration, as for instance in running or on exertion.

(4.) We have in the respiratory undulations of the Traube-Hering curves very strong evidence that the bronchi contract and dilate rhythmically in the manner suggested. The arterial pressure undergoes regular rhythmical variations, owing to the rhythmical action of the respiratory centre on the vaso-motor centre. We have then experimental evidence that while the respiratory centre is rhythmically sending the impulses of inspiration and expiration, the neighbouring vaso-motor and cardio-inhibitory centres are acting in unison, so that their influence on the arterial tension and the heart respectively waxes and wanes rhythmically with each respiratory movement.

CAROTID BLOOD-PRESSURE TRACING OF A DOG. (Stirling.)

I = commencement of inspiration; E = commencement of expiration. The respiratory undulations are due in part, at any rate, to a stimulation of the vaso-motor centre, which runs parallel with the respiratory movements, and which causes the arteries to contract, with consequent raising of the arterial pressure. The smaller number of pulse-beats during the greater part of expiration is due to the activity of the cardio-inhibitory centre in the medulla, this centre being comparatively inactive during inspiration.

N.B.—Dog under simple anæsthesia without artificial respiration, and vagi not divided.

Now, inasmuch as we have evidence in the respiratory undulations in Traube-Hering curves that the respiratory centre actually induces rhythmical *vaso-motor* impulses through a *neighbouring* centre, we have very good reason to believe that it induces a corresponding *broncho-motor* influence *in its own particular territory*, and the evidence on this point becomes almost conclusive when we observe the influence of the respirations on the laryngeal centre is such as to cause rhythmical abduction and adduction of the vocal cords.

(5.) At birth, before respiration has occurred, the lungs are solid and in an atelectatic condition, and at the first breath the air inspired has to overcome the adhesion of the surfaces of the bronchial mucosa and of the alveolar epithelium. It is only after some time has elapsed that the lung is fully expanded; and while expiration with a partially-closed glottis, as in crying, must of course aid in the alveolar dilation, the pulmonary expansion, which occurs in spite of the tendency of the lungs to contract, is probably aided by the bronchial contraction during expiration, otherwise the chest would tend to remain collapsed if the air passed out of the bronchial tubes just as readily as it entered.

I submit there is reason to believe that, in children, the bronchioles dilate and contract rhythmically with inspiration and expiration, while in adults the expiratory contraction may be in abeyance during quiet respiration. That in deep inspiration and expiration, both in adults and in children, the expiratory afferent impulses are more pronounced, and inspiration and expiration are associated with bronchiole dilatation and contraction.

That in so-called bronchial spasm of asthma and acute bronchitis the bronchi dilate and contract as in normal infantile respiration, but that the contraction phase is excessive and prolonged.

That excessive bronchial expiratory contraction is the cause of emphysema in asthma and in bronchitis, and that chronic hypertrophous emphysema is similarly induced.

## Figures and Tables

**Figure f1:**